# Supplementary figures and images for: Improved detection of air trapping on expiratory computed tomography using deep learning
Source: PLoS One. 2021 Mar 24;16(3):e0248902. doi: 10.1371/journal.pone.0248902 (PMC7990199; doi:10.1371/journal.pone.0248902)

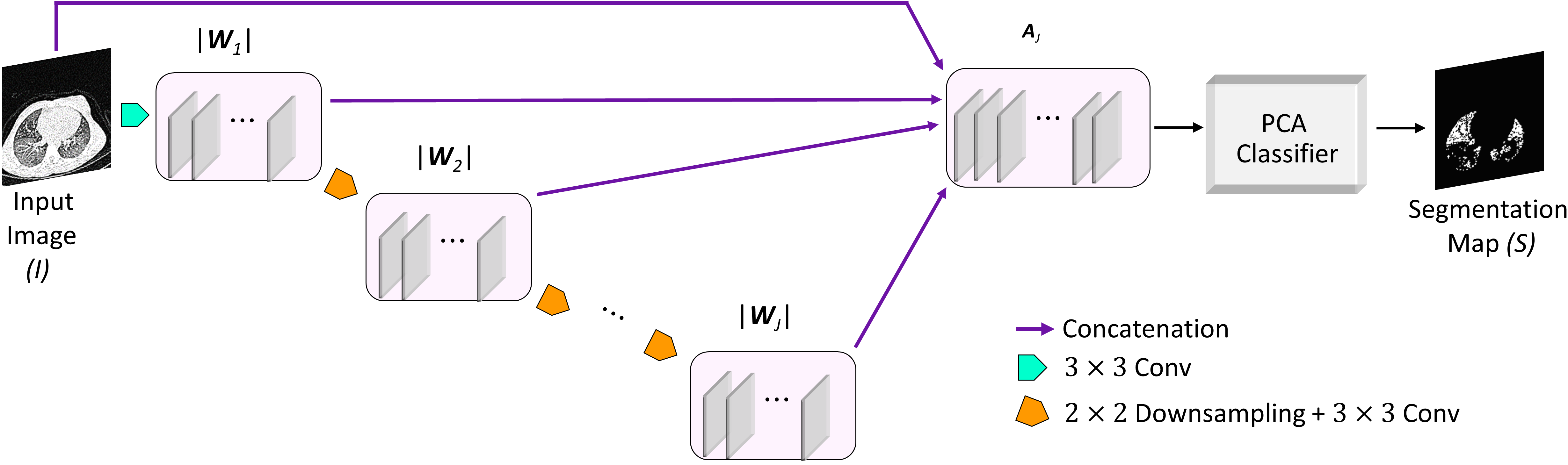

Supplement: S1 Fig — Schematic overview of the scattering convolutional network (SN) architecture, where the |WJ|’s represent the modulus wavelet transform at each scale J, and AJ concatenates the averaged signals (the detailed coefficients) of the wavelet transform at all scales. We used a total of J = 4 scales in our implementation. (PNG) [file pone.0248902.s001.PNG]

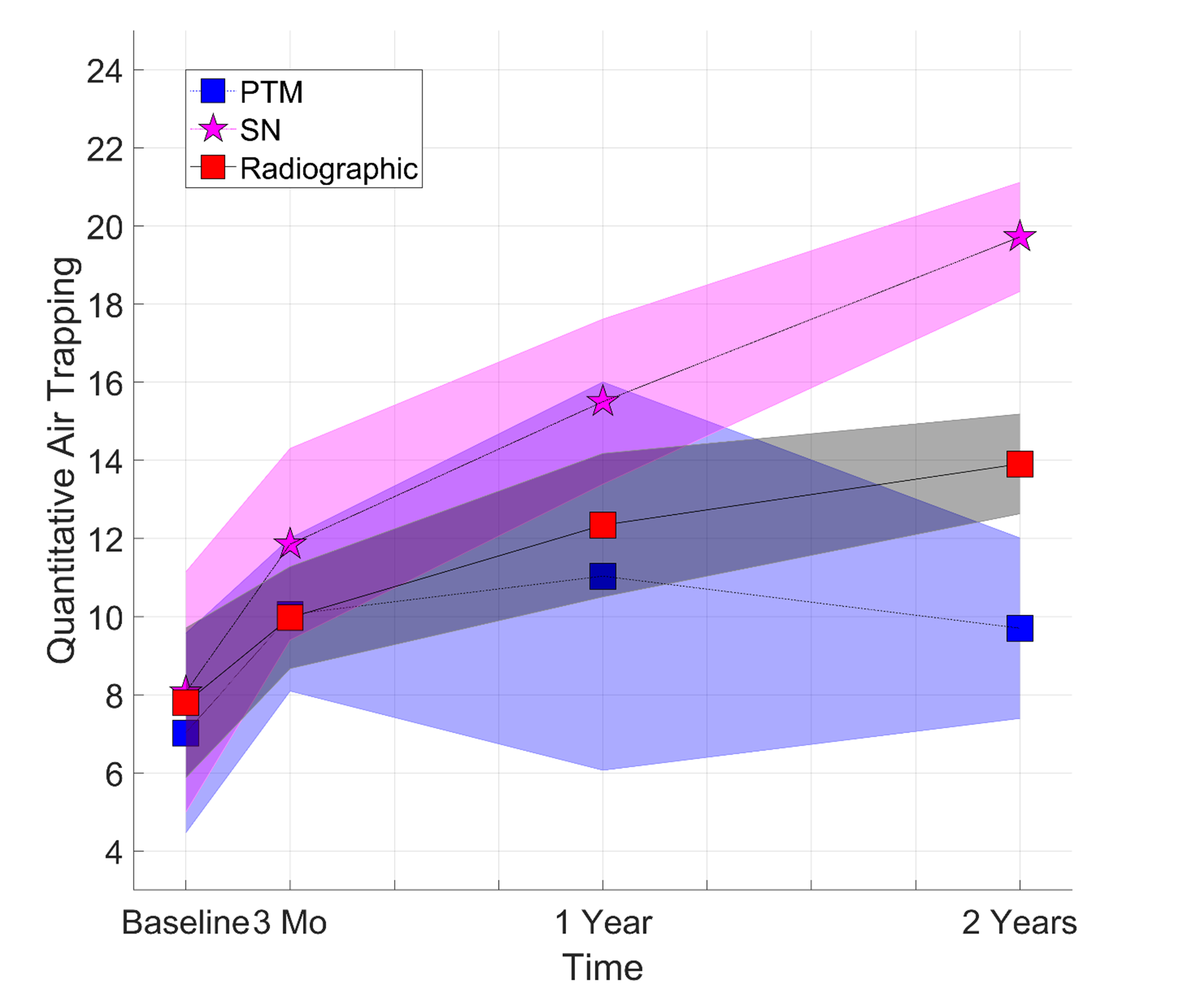

Supplement: S2 Fig — Presented are Quantitative air trapping (QAT) results of the various methods from Site 1 cohort at the different examination times. (PNG) [file pone.0248902.s002.PNG]
