# Supplementary material for: Improved detection of air trapping on expiratory computed tomography using deep learning
Source: PLoS One. 2021 Mar 24;16(3):e0248902. doi: 10.1371/journal.pone.0248902 (PMC7990199; doi:10.1371/journal.pone.0248902)
Supplement: S1 Appendix — (DOCX) [file pone.0248902.s003.docx]

**Supplementary Material**

**SUPPLEMENTARY METHOD 1**

**Scattering Convolutional Network (SN)**

To confirm that the results observed using our proposed DN method did not introduce any bias, we trained and tested a separate CNN model referred to as the scattering convolutional network (SN). The SN model was based on a scattering transform defined by Bruna and Mallet in [1]. The proposed SN consists of a total of four scales ($\boldsymbol{J}= 4$) where at each scale we employ a Gabor wavelet ($\boldsymbol{W}$) with five different orientation filters ($K = 5$) separated by $\pi k/K$ angles, where $k = \left\{ 1,\cdots,K \right\}$. The scattering coefficients at each scale were obtained by a wavelet transformation, followed by a non-linear pointwise complex modulus applied to the wavelet coefficients, a local averaging operator $\boldsymbol{A}_{J}$, and downsampling of scale $2^{J}$. Once all scattering coefficients were obtained, they were concatenated together and used as features for classification. We trained the model using a generative principal component analysis (PCA) classifier using the features from SN to identify the areas of AT on the CT images. A schematic representation of the proposed SN is shown in **S1** **Figure**.

*Training*

The SN was trained to minimize the Dice loss, similar to that of DN. Similar to the DN model a nested 2-fold cross-validation strategy was employed for training the SN model. Although, we used the same strategy for training both the SN and the DN model using the data from the Site 1 of 96 images (N = 24; with four different time points), the training and test data were randomly selected for each model separately. The SN architecture was implemented in MATLAB (version 2019a, MathWorks, Natick, Mass). The filters in the SN architecture are fixed Gabor wavelet filters at different orientations. The final layer in the SN architecture was trained using a PCA classifier as described in [1]. The SN was trained on a workstation different from that of the DN method running a 64-bit Windows operating system (Windows 10) with an Intel Xeon W-2123 CPU at 3.6GHz with 64GB DDR4 RAM and an NVIDIA GeForce RTX 2080 graphic card with 2944 CUDA cores (Nvidia driver 411.63) and 12GB GDDR6 RAM.

**SUPPLEMENTARY RESULT 1**

**Comparison of QAT Measurements**

The SN model was found to detect air trapping that increased in a time dependent manner similar to the DN model. At baseline, good agreement was observed for the QAT_SN_ to QAT_PTM_ with a difference in QAT values of about only 1% (**S2 Fig.**). Evaluating the QAT values over time, we saw a poor agreement between the SN QAT values to the QAT_PTM_ as the SN model detected increasing amounts of AT over the two-year period (**S2 Fig.**) like that of the DN model in **Fig. 3**). The QAT_SN_ significantly increased from baseline to year two of the trial by up to 11.6% ± 1.7%. The QAT_SN_ measurements post-baseline examination were also significantly different with a *p*-value of 0.03 between the intervals of 3 to 12 months and a *p-*value of 0.002 between the intervals of 12 to 24 months, respectively.

**Supplemental References:**

1. Bruna J, Mallat S. Invariant scattering convolution networks. IEEE Trans Pattern Anal Mach Intell. 2013;35(8):1872-86.
